# Retraction Note: Novel role of miR-133a-3p in repressing gastric cancer growth and metastasis via blocking autophagy-mediated glutaminolysis

**DOI:** 10.1186/s13046-024-03123-7

**Published:** 2024-07-18

**Authors:** Xing Zhang, Zheng Li, Zhe Xuan, Penghui Xu, Weizhi Wang, Zheng Chen, Sen Wang, Guangli Sun, Jianghao Xu, Zekuan Xu

**Affiliations:** 1 Department of General Surgery, The First Affiliated Hospital of Nanjing Medical University, No.300, Guangzhou road, Nanjing, Jiangsu province China; 2 Department of Surgical Oncology, University of Miami, Miami, USA; 3 Collaborative Innovation Center For Cancer Personalized Medicine, Nanjing Medical University, Nanjing, 210029 Jiangsu Province China

**Retraction Note:** ***J Exp Clin Cancer Res*** **37, 320 (2018)**

10.1186/s13046-018-0993-y

The Editor-in-Chief has retracted this article. After publication, concerns were raised regarding the data presented in some of the figures, specifically:

- Figure 2C BGC-823 miR-NC images appear to overlap with Fig. 7C MKN45 LV-miR-IN + si GLS.
- Figure 2D BGC-823 NC images appears highly similar to Fig. 5A MKN-45 miR-133a-3p IN+, HCQ+.
- Figure 2E MKN-45 Invasion miR-133a-3p mimic image appears to overlap with BGC-823 Invasion miR-133a-3p mimic.
- Figure 2E BGC-823 Migration miR-133a-3p mimic images appears to overlap with Fig. 7D BGC-823 Migration LV-miR-IN + BPTES.
- Figure 2F miR-133a-3p mimic Day10 image appears highly similar to Fig. 5E circNRIP1 ov day1 in [1].
- Figure 5D invasion miR-IN + si GABARAPL1 and miR-IN + HCQ images appear to overlap.
- Figure 10F GLS LV-miR-133a-3p image appears to overlap with GDH LV-miR + HCQ + BPTES.

The Editor-in-Chief therefore no longer has confidence in the presented data.

Xing Zhang, Zheng Li, Zhe Xuan, Penghui Xu and Zekuan Xu agree to this retraction. Weizhi Wang, Zheng Chen, Sen Wang, Guangli Sun and Jianghao Xu have not responded to any correspondence from the editor or publisher about this retraction.
